# Employee accountability in Indonesia: The role of formalization, managerial monitoring behavior and perceived competence

**DOI:** 10.1371/journal.pone.0278330

**Published:** 2022-12-16

**Authors:** Deri Natria, Samian, Corina D. Riantoputra

**Affiliations:** 1 Faculty of Psychology, Universitas Indonesia, Depok, West Java, Indonesia; 2 Faculty of Psychology, Universitas Airlangga, Surabaya, East Java, Indonesia; Universiti Pertahanan Nasional Malaysia, MALAYSIA

## Abstract

Accountability is an imperative element of organizations that Human Resource Management establishes as a behavior guideline. It encourages employees to be responsible for decisions and actions they took. Employing Social Cognitive theory, this study aims to investigate the mechanism of how formalization influences employee accountability. We hypothesize that this relationship is mediated by managerial monitoring behavior and perceived competence. Data was collected from 331 employees of a government agency in Indonesia. Structural equation modelling analysis reveals that (1) formalization directly and indirectly influences employee accountability, and its direct effect is higher than its indirect effect, suggesting the importance of formalization system in Human Resource Management; (2) the contribution of perceived competence as a mediator between formalization and employee accountability is higher than the contribution of managerial monitoring behavior, suggesting the importance of micro-foundations of Human Resource research; (3) the relationship between formalization and employee accountability was serially mediated by managerial monitoring behavior, which was preceded by managerial monitoring behavior for task performance and continued by managerial monitoring behavior for interpersonal facilitation This study contributes to international Human Resource Management literature by explaining the mechanism by which formalization affect accountability.

## Introduction

Accountability is a fundamental concept that influences the perceptions, behavior, and social dynamics of employees in the daily life of organizations [1]. It encourages individuals to be responsible for their decisions and actions in realizing the social order. While, 91% of employees agree that accountability is essential in organizations, 82% of managers admit they have limited to no ability to hold their employees accountable [2]. This data suggests that while, in theory, the importance of accountability is accepted, in reality, much needs to be done to understand and improve accountability in the workplace. Employee accountability refers to “perceived expectation that one’s decisions or actions will be evaluated by a salient audience and that rewards or sanctions are believed to be contingent on this expected evaluation” [3, p.134]. In other words, this understanding is based on the individual’s perception of accountability as opposed to the attributions of accountability that an audience imposes [4]. Essential in this definition is the expected evaluation, requirement for account giving, consequences for the potential evaluation and salient audience for the behavior. When an accountable actor knows he or she will receive a reward or sanction from the relevant audience (e.g., leaders, regulators, organizations), the actor may try to meet the expectations of the audience that conducted the evaluation [4]. Employee accountability also helps employees stay motivated and do their jobs, while also controlling their behaviors to ensure that the organization can run effectively [3]. It can be concluded that employee accountability acts as a guideline for behavior in society, and a classic and importance challenge for organizations to face [5].

Previous studies have begun to discover the effects of employee accountability. For example, Hochwarter et al. [6] and Laird et al. [7] found that employee accountability has an indirect influence on employee performance and job satisfaction. Employee accountability is also known to influence behavior within teams [8, 9], decision-making [10, 11], and turnover intention [1]. Meanwhile, research which examines the impact of accountability in the context of Human Resource Management (HRM) in the US federal government by Han and Hong [12] found that accountability manifested in the HRM function positively affects organizational performance. Wang et al., [13], in their research on in Chinese government agencies, found that employee accountability was formally realized and enforced through the HRM system, where the organization manages and monitors employee behavior. These findings indicate that employee accountability is an important variable for organizations to consider.

In addition to its impact, researchers have just begun to examine the antecedents of employee accountability, both internally and externally. Internal factors that affect employee accountability includes affective trait [14], core-self evaluation [15], and attribution style [1]. Researchers have also found several external factors to be antecedents of employee accountability. For example, leadership factors [15–17] and organizational factors, such as organizational structure and organizational culture, are known to be external factors that play important roles in employee accountability [14, 16, 18, 19].

This article aims to fill in several research gaps. First, the role of formalization on employee accountability. Formalization refers to “the design parameter by which work processes are standardized, through rules, procedures, policy manuals, job descriptions, work instructions, and so on” [20, p.325]. It is a part of organizational factors that have been minimally studied in relation to employee accountability. Dewi and Riantoputra [14] discussed the potential for a positive relationship between formalization and employee accountability, especially in government agencies that tend to create many rules, regulations, and procedures to regulate employee behavior (high formalization). Formalization encourages employees to act in accordance with certain rules, procedures, and instructions, so as not to deviate from the standardized organizational work processes [21]. This happens because standard work procedures created through formalization reduce role ambiguity and can help employees understand the expectations of organizations and leaders [22]. The existence of formalization provides clarity to employees regarding what should or should not be done, as well as regarding the limits of authority and to whom employees are responsible for their actions and decisions. Therefore, further explanation is needed about the important role of formalization in the formation of employee accountability.

Second, there is a lack of understanding about the mechanism by which formalization affects employee accountability. Hall et al. [4] indicated that organizations who expect their employees to be accountable need to ensure that their leaders take a role in increasing employee accountability. This is further supported by Dewi and Riantoputra [14], who suggested the need to examine leadership factors as one of the antecedents of employee accountability because leaders create rules and systems to motivate subordinates and provide them with a model to act with accountability. As noted by Lambert et al. [23], formalization provides guidance, especially for leaders, to direct and respond to their subordinates. Moreover, leaders are considered representatives of the organization [24], and, as such, they can give instructions and examples of work rules, as well as organizational support to their subordinates. Through managerial monitoring behavior, it is argued that leaders will be able optimilized the relationship between formalization and employee accountability. Managerial monitoring behavior is defined as “a form of direct supervision that considers the extent to which the manager engages in administrative behaviors that reinforce perceptions of accountability in the manager’s employees” [17, p.1630]. Using managerial monitoring behavior, leaders provide important cues to employees that clarify their tasks, while strengthening obligations and personal control over behaviors and outcomes that are important for the organizations. These cues communicate the goals that the leaders expect to achieve and how employees are expected to contribute to achieving those goals, as well as benchmarks for performance success. In so doing, these cues support the development of employee accountability. In this study, we focus on two domains of managerial monitoring behavior. The first domain focuses on the perceive importance of task performance (managerial monitoring behavior for task performance; MMBTP), whereas the second domain focuses on the perceived importance of helping and cooperating with other employees (managerial monitoring behavior for interpersonal facilitation; MMBIF).

Third, differ from Mero et al. [17], who demonstrated that MMBTP and MMBIF, parallelly, related with employee accountability, this study contends that MMBTP and MMBIF mediate the relationship between formalization and employee accountability, serially. As shown by Josephine and Riantoputra’s [25] study on 85 pairs of superiors and subordinates in private companies in Indonesia, MMBTP is not directly related to employee accountability, whereas MMBIF is. Further, Harzer and Ruch [26] explain that interpersonal facilitation is the act of engaging in interpersonally oriented behaviors that are necessary for completing tasks in organizational settings. As a result, we argue that, MMBTP and MMBIF may sequentially mediated the relationship between formalization and employee accountability.

Fourth, this article aims to fill in the research gap in relation to how an individual factor (i.e., perceived competence) mediates the relationship between formalization and employee accountability. Research has shown that perceived competence is a powerful individual factor influencing work behavior [27]. Perceived competence refers to “an individual’s belief in his or her capability to perform activities with skill” [28, p.472]. It is one of the concepts of self-efficacy that is particularly relevant to work roles [27]. In their research, Hall et al. [29] revealed that there is a significant and positive relationship between perceived competence and employee accountability, while Hempel et al., [30] and Rhee et al., [31] demonstrated a significant relationship between formalization and perceived competence. Altogether, their research results encourage us to examine the mediating role of perceived competence on formalization and employee accountability. We argue that high formalization, which can be seen through clear work boundaries and responsibilities, may increase perceived competence; and then perceived competence may increase employee accountability, because employees become more accountable for what they do when they have greater confidence in their abilities.

In brief, this study aims to investigate whether managerial monitoring behavior and perceived competence mediate the relationship between formalization and employee accountability. This research question will be explored using social cognitive theory (SCT), which explains how individuals in social systems apply various processes, including the process of acquiring and adopting knowledge, with a main focus on the learning process and the interrelation of various factors within it. SCT involves three interacting components: person, environment and behavior [32]. The interaction between components is explained by SCT through the abilities of symbolizing, forethought, vicarious learning, self-regulation, and self-reflection. This theory assumes that most of employees’ knowledge and behavior are also formed by the organizational environment in which they work [33].

SCT refers to the dynamic interaction and reciprocal relationships between person, environment, and behavior [32]. In this model, the three components operate as determinants that reciprocally influence each other. However, these components do not influence each other equally, nor simultaneously. Rather, there is a time delay for the causal factors to influence other factors or reciprocate influences from other factors. In this study, we build upon SCT to explain the interactions between the proposed variables of formalization, managerial monitoring behavior, perceived competence, and lastly employee accountability.

SCT explains the nature of two-way mutual influence through five basic human abilities: (1) symbolizing, (2) forethought, (3) vicarious learning, (4) self-regulation, and (5) self-reflection. This theory shows that humans have extraordinary symbolizing abilities that allow them to successfully react and then change and adapt to their respective environments. Through symbolizing, people also assume the meaning, form, and duration of their past experiences. Employees initiate and guide their actions in an anticipatory manner through forethought. Employees’ capacity for vicarious learning allows them to acquire and collect rules for initiating and controlling different patterns of behavior without having to acquire these behaviors gradually through risky trial and error. Self-regulation has a central role in SCT, as it indicates that a person’s behavior is not always in accordance with the standards given by others but, rather, is based on the standards of the individual. Meanwhile, self-reflection allows people to think about and analyze their experiences and thought processes. Employees use these basic abilities to initiate, regulate, and maintain their own behavior.

### Formalization and employee accountability

In organizations with high formalization, employees are prompted to follow procedures and rules while carrying out their work [34]. According to Hempel et al. [30], the existence of these procedures and rules allows team members to identify areas and decisions that are within the scope of their responsibilities. Formalization also allow employees’ behavior to be more predictable because their work processes (e.g., coordinations and communications) are governed within standardized parameters [35]. For example, if problems arise in the workplace, a formalized procedure will help employees to consistently and effectively solve the issues. Furthermore, formalization facilitates active employee engagement and emphasizes a cooperative management style [36]. Formalization also relieves role stress and helps individuals to be more effective by providing guidance and clarifying responsibilities [37]. In a similar way, formalization can also help clarify the audience’s expectations with regard to employee accountability.

**Fig 1:**
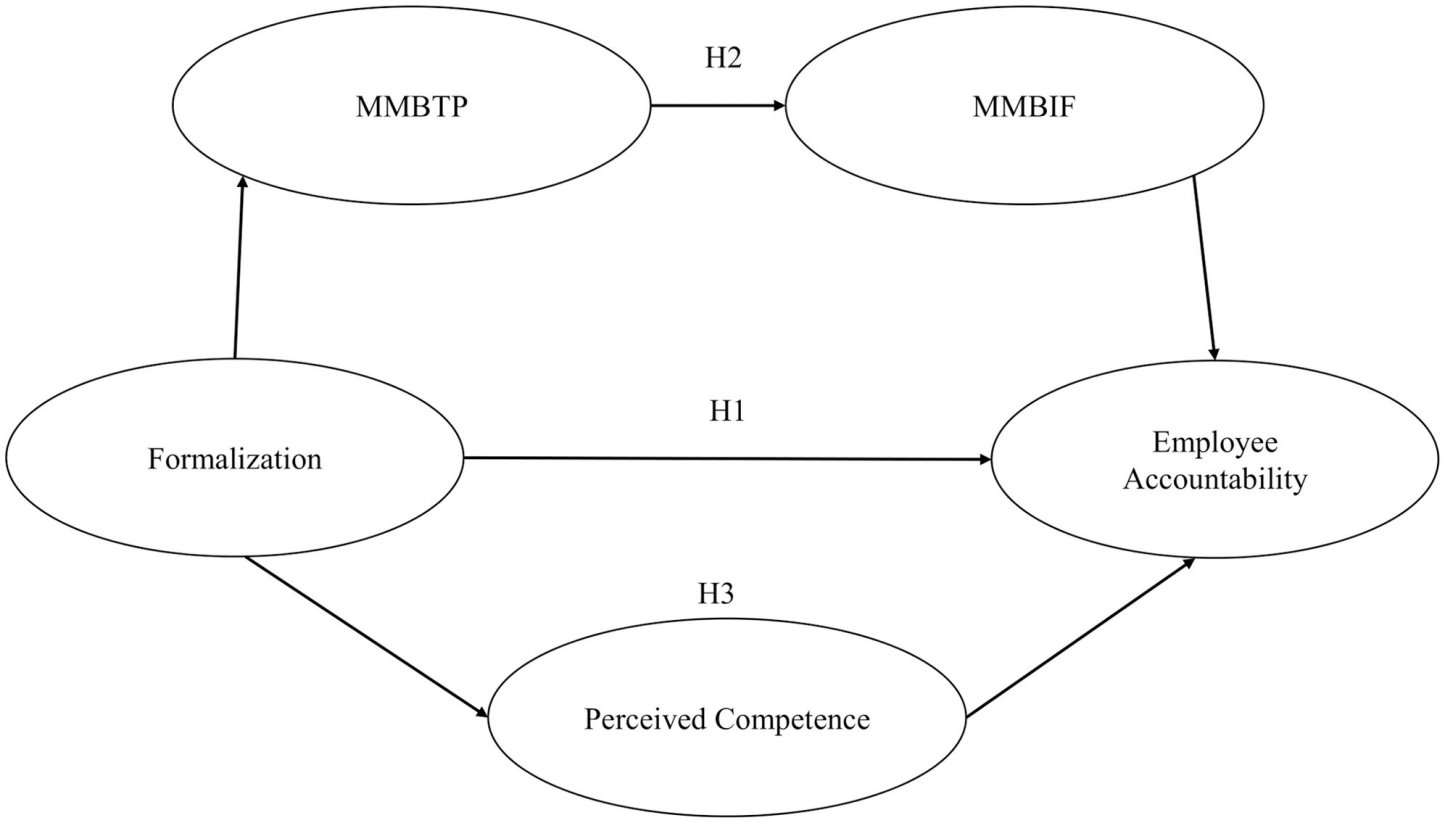
The research model of the study. MMBTP = Managerial monitoring behavior for task performance; MMBIF = Managerial monitoring behavior for interpersonal facilitation.

SCT explains the relationship mechanism of formalization and employee accountability regarding how individuals in the social system use a variety of techniques, with the learning process and the interactions of its numerous components as the primary considerations. Wood and Bandura [32] also mention that human behavior is extensively regulated by the effects of the behavior, such that individuals motivate and regulate their behavior based on consequences (self-regulation). SCT explains that the environment shapes and controls individual behavior. In this case, formalization as organizational control provides a reference for good and accountable behavior to the organizational members. Formalization allows employees to know what they can or cannot decide and to identify areas of responsibility for the team [38]. Thus, we hypothesize (see Fig 1):

HI: Formalization has a significant and positive relationship with employee accountability.

### Managerial monitoring behavior as mediator

Leaders manifest organizational control toward their employees by clarifying important organizational tasks and monitoring their behaviors. In a study conducted by Lambert et al. [23] with 827 police officers in India, a higher level of formalization was related to greater employee engagement, satisfaction, and commitment. Furthermore, they also maintained that a high level of formalization could help regulate the relationship between leaders and their subordinates, such as by constraining leaders’ harmful or complacent behaviors. Additionally, Krasman [39] also showed that formalization was related to employees’ trust in their leaders.

Employees will acquire superior behavior through symbolizing abilities in the three components of SCT idea. As leaders engage more frequently in monitoring behavior (e.g., by asking questions or inquiring about progresses), employees become increasingly aware and perceptive of the importance of the outcomes. This is also suggested by Mero et al. [17], who contended that, by monitoring behavior and work outcomes, leaders get the opportunity to provide performance feedback for their subordinates and help them prioritize tasks. It is important to note that there are two domains of managerial monitoring behavior [17]. The first of these is managerial monitoring behavior for task performance (MMBTP). According to Guidice et al. [16], in MMBTP, leaders focus on monitoring subordinates’ task performance, which is necessary to support the organization’s business processes. Employee behaviors within the task performance domain are usually recognized as formal requirements of their job description and performance appraisal, as task performance is the technical core of the organization. Leaders with adequate MMBTP skills will ensure that subordinates know their responsibilities and are motivated to do their jobs. The second domain of managerial monitoring behavior is the interpersonal facilitation domain (MMBIF). In MMBIF, leaders supervise their subordinates’ involvement in cooperating with, considering, and helping other employees [40]. Moreover, interpersonal facilitation consists of interpersonally oriented behaviors that contribute to the achievement of organizational goals. Interpersonal facilitation correlates with teamwork, kindness, leadership, and fairness [26]. It can help reduce conflict, help improve cooperation, and help employees deal with problems that affect their performance. It should be noted that both domains are important in allowing leaders to identify employees who are ‘star’ performers within their organizations [41]. The findings of Min et al’s [42] research also demonstrate that managerial behavior (person-oriented skills and task-oriented skills) will influence employee performance (task performance and interpersonal facilitation).

SCT assumes that the organizational setting shapes employee knowledge and behavior [33]. Within the organizational setting, leaders are regarded as the decision makers. Tetlock and Lerner [43] mentioned that employee accountability interacts with the characteristics of the decision maker and the nature of the task environment in order to create a beneficial impact. Leaders implement organizational control systems to their employees by clarifying important organizational tasks and goals and controlling behavior through managerial monitoring. The feedback control system is one of the processes involved in the formation of self-regulation in the context of SCT.

Furthermore, Goodyear [44] suggested that level of interaction between audience and actor correlates with the level of accountability of the actors. Based on this assumption, we argue that employees tend to be more accountable when the audiences of their action are leaders, their institutions, or government agencies. That is, formalization directs leaders in monitoring how employees conduct their tasks and work procedures. In other words, formalization influences the frequency and intensity of managerial monitoring behavior related to task performance (MMBTP) which is then followed by managerial monitoring behavior related to how employees support each other for competing their tasks. Previous research in Indonesia demonstrated that only MMBIF significantly related to accountability [25]. Therefore we argue that MMBTP influences the intensity of MMBIF, which help employees in their self-regulation to be accountable in their job. Thus, we hypothesize (see Fig 1):

H2: Managerial monitoring behavior for task performance and managerial monitoring behavior for interpersonal facilitation, serially, mediate the relationship between formalization and employee accountability.

### Perceived competence as mediator

The relationship between formalization and employee accountability is mediated not only by managerial monitoring behavior but also by perceived competence. In light of the interaction of the three components of SCT (i.e., person, behavior, and environment), perceived competence is an individual cognitive factor that is affected by the environment. Formalization is an important aspect of the work environment that can influence how employees feel about their ability to complete work and how that perception translates into work behavior. Research conducted by Hempel et al. [30] on team empowerment and organizational context among 94 Chinese high-tech companies supports the role of formalization in promoting perceived competence. Formalization can strengthen perceived competence by clarifying job roles, improving job performance, and organizing cooperation and collaboration among employees.

Given the relationship between formalization and perceived competence, we argue that the relationship between formalization and employee accountability may also be mediated by perceived competence. Hall et al. [29] revealed that perceived competence has a significant and positive impact on employee accountability. This is in line with Spreitzer’s [27] findings, which showed that perceived competence influenced the work behavior of 393 managers from various Fortune 50 organizations. Employees’ belief in their competence is influenced by mastery experiences, vicarious learning, social persuasion, and affective states. If employees lack mastery experiences, role models in their workplace, or persuasion from their leaders or colleagues, their beliefs in their competence will be weaker [45], leading them to doubt their competence and damaging their performance levels.

As discussed earlier, according to SCT, the nature of the bidirectional reciprocal influences can be explained using five basic human capabilities (symbolizing, forethought, vicarious learning, self-regulation, and self-reflection). These capabilities are essential for employees to initiate, organize, and maintain their own behavior. Furthermore, these capabilities can help explain differences in employee behavior, especially those caused by differing levels of perceived competence within the same organizational settings [37]. Chamberlin et al. [46] demonstrated that using SCT allowed them to understand that employees will be more engaged when they believe in their own abilities, have a sense of competence in their skills, and feel a sense of control over their environment. Therefore, we hypothesize:

H3: Perceived competence mediates the relationship between formalization and employee accountability.

## Methods

### Participants and procedures

The study was conducted at a government agency with headquarters in Jakarta and representative offices spread across 34 provinces in Indonesia. We chose this particular government agency because they engaged in financial examination activities, which often stress a high level of accountability. This is further supported by the fact that the government agency also implements regular internal employee rotation to maintain integrity and accountability. Moreover, we argue that formalization plays an important role in a governmental agency’s governing of its employees. To gain a comprehensive picture of employee accountability in this organization, we set the population of this study as all of the employees from both the headquarters and its representative offices.

Data were collected through online surveys utilizing the SurveyMonkey platform. The surveys were distributed with permission from the intended government agency. We employed a convenience sampling technique because of its ease of use. Additionally, respondents must have had a working period of at least one year and have worked under their direct supervision for at least one year. This is important because respondents needed to assess the formalization of the organization and managerial monitoring behavior of its leaders in the survey.

Initial research data were obtained from 388 respondents. However, we only analyzed 331 respondents who had completely filled in the surveys and matched our respondent criteria. A total of 52.3% of the respondents were men and 55.9% had a Bachelor’s degree. The average respondent age was 37.68 years old (SD = 6.75), and the average job tenure was 12.43 years (SD = 5.65) (see Table 1 below).

**Table 1:** Demographic data.

Characteristic		Frequency	Percentage
**Gender**	Male	173	52.3%
	Female	158	47.3%
**Job Level**	Staff	287	86.7%
	Echelon 4	24	7.3%
	Echelon 3	20	6.0%
**Education**	High School	10	3.0%
	Vocational	9	2.7%
	Undergraduate	185	55.9%
	Post graduate	127	38.4%

N = 331

### Ethical approval

This study protocol was evaluated and approved by the Ethics Committee Team of the Faculty of Psychology, Universitas Indonesia (approval number. 121/Fpsi.Komite Etik/PDP.04.00/2021). Respondents were informed prior to the survey regarding the purpose of research and were given assurance for the confidentiality of data. Participation was completely anonymous and voluntarily. All respondents provided their informed consent electronically by clicking in a button with the following text: “I agree to participate in this survey”.

### Measures

Each measure was translated into *Bahasa Indonesia* (Indonesian Language) through a back-translation process and employed a 6-point Likert scale to record individual responses. *Employee accountability* was measured using the eight-item scale adapted from Hochwarter et al. [47], with each item rated on a 6-point Likert scale (1 = *strongly disagree*, 6 = *strongly agree*). One example item is “Co-workers, subordinates, and leaders closely scrutinize my efforts at work” (Cronbach alpha = 0.70).

*Formalization* was measured with a five-item scale adapted from Pugh et al. [48], which asked the respondents to assess the extent to which their work environment was governed by rules and procedures (1 = *strongly disagree*, 6 = *strongly agree*). An example item is “There are written guidelines and procedures available in this organization” (Cronbach alpha = 0.77).

*Managerial monitoring behavior for task performance* was measured by employing six items from the ‘accountability for task’ dimension in Mero et al.’s [17] Managerial Monitoring Behavior scale. Item response options range from 1 (*never*) to 6 (*always*). A sample item is “In the past year, how often your leader asked your progress on a task activity” (Cronbach alpha = 0.91).

*Managerial monitoring behavior for interpersonal facilitation* was measured using six items adapted from the ‘accountability for interpersonal facilitation’ factor in Mero et al.’s [17] Managerial Monitoring Behavior scale. Item response options range from 1 (*never*) to 6 (*always*). An example item is “In the past year, how often your leader asked your activities related to helping and cooperating with others at work” (Cronbach alpha = 0.96).

*Perceived competence* was assessed using five items from Spreitzer [27]. Respondents were asked to rate their abilities in their respective work role on 6-point Likert scale (1 = *strongly disagree*, 6 = *strongly agree*). A sample item is “I am confident about my ability to do my job” (Cronbach alpha = 0.96).

### Analytical technique

The data in this study were analyzed using Structural Equation Modeling (SEM), which was processed using IBM SPSS AMOS 24. Item parceling was performed to reduce the possibility of psychometric problems, stabilize parameter estimates, and improve model fit [49]. Compared to individual items, parceled items also have a better property distribution [50].

## Results

An initial Harman’s Single Factor Test was employed to detect common method bias, and the result yielded a value of 29.26%. This result indicated that common method bias was not present in this study, and thus it was not considered as a problem [51].

The consistency of respondents’ responses to the survey questionnaire was checked in this study by evaluating the reliability of measurement items. Our study used Cronbach’s alpha to assess the reliability of five constructs and discovered that Cronbach’s value is greater than 0.7 for all constructs. Convergent validity was also evaluated. The ideal standardized average variance extracted (AVE) should be greater than 0.5, and reliability must be greater than 0.7 to demonstrate sufficient convergent validity [52]. The results of reliability and validity are shown in Table 2.

**Table 2:** Construct reliability and validity.

Main Variables	Cronbach’s Alpha	Composite Reliability	Average Variance Extracted (AVE)
Formalization	0.77	0.82	0.54
MMBTP	0.91	0.85	0.51
MMBIF	0.96	0.95	0.65
Perceived Competence	0.96	0.90	0.69
Employee Accountability	0,70	0.87	0.54

MMBTP = Managerial Monitoring Behavior for Task Performance; MMBIF = Managerial Monitoring Behavior for Interpersonal Facilitation

We performed confirmatory factor analysis (CFA) for all variables under study and evaluated the construct validity. Sun [53] recommended standardized root mean square residual (SRMR), comparative fit index (CFI), root mean square error of approximation (RMSEA), and Tucker-Lewis index (TLI) as the rules of thumb to evaluate the construct validity. As shown in Table 3, the five-factor model best fits the data, recommending support for variable distinctiveness.

**Table 3:** Confirmatory factor analysis.

Model	χ^2^	df	χ^2^/df	SRMR	CFI	RMSEA	TLI
Four-factor model^a^	1547	399	3.88	0.08	0.81	0.09	0.79
Five-factor model^b^	1178	395	2.98	0.08	0.87	0.08	0.86

SRMR = standardized root mean square residual; CFI = comparative fit index; RMSEA = root mean square error of approximation; TLI = Tucker-Lewis index;

^a^Formalization, managerial monitoring behavior, perceived competence, employee accountability;

^b^Formalization, managerial monitoring behavior for task performance, managerial monitoring behavior for interpersonal facilitiation, perceived competence, employee accountability

Means, standard deviation, and correlations are shown in Table 4. Table 4 show that there is a significant relationship between variables in the model.

**Table 4:** Mean, standard deviation, and correlation.

		**Mean**	**SD**	**Gender**	**Age**	**Job level**	**Education**	**Job tenure**	**MMBTP**	**MMBIF**	**PC**	**Fo**	**EA**
1	Gender	-	-	1									
2	Age	37.68	6.75	0.00	1								
3	Job level	1.19	0.53	-0.02	0.46**	1							
4	Education	-	-	-0.08	0.25**	0.29**	1						
5	Job tenure	12.43	5.65	0.03	0.83**	0.52**	0.25**	1					
6	MMBTP	3.94	0.99	-0.07	0.05	0.15**	0.05	0.07	1				
7	MMBIF	3.55	1.09	-0.10	0.02	0.18**	0.06	0.03	0.75**	1			
8	PC	4.94	0.53	-0.12*	0.09	0.22**	0.16**	0.10	0.11*	0.09	1		
9	Fo	4.49	0.74	-0.05	0.16**	0.14*	0.12*	0.17**	0.20**	0.21**	0.34**	1	
10	EA	4.36	0.61	-0.09	0.07	0.14**	0.12*	0.10	0.20**	0.25**	0.38**	0.32**	1

* p< 0.05;

** p<0.01;

MMBTP = Managerial Monitoring Behavior for Task Performance; MMBIF = Managerial Monitoring Behavior for Interpersonal Facilitation; PC = Perceived Competence; Fo = Formalization; EA = Employee Accountability

The model fitness indices for our hypothesized SEM model yields χ^2^ = 105.22, df = 44, χ^2^/df = 2.39, RMSEA = 0.07, TLI = 0.96, CFI = 0.97, AGFI = 0.91, GFI = 0.95. A fit model is indicated by RMSEA values less than or equal to 0.08 [54]. If the TLI and CFI values are closer to one, then the model has a good fit [55]. Kline [56] states that the fit value of χ^2^/df is less than three. So it can be concluded that this research model has a good fit [57]. The value of R^2^ shows how much the latent variable is influenced by the predictors. The greater the R^2^, the greater the influence obtained. The R^2^ value of employee accountability is 0.32, meaning that 32% of employee accountability is influence by MMBTP, MMBIF, and perceived competence by controlling the job level.

**Fig 2:**
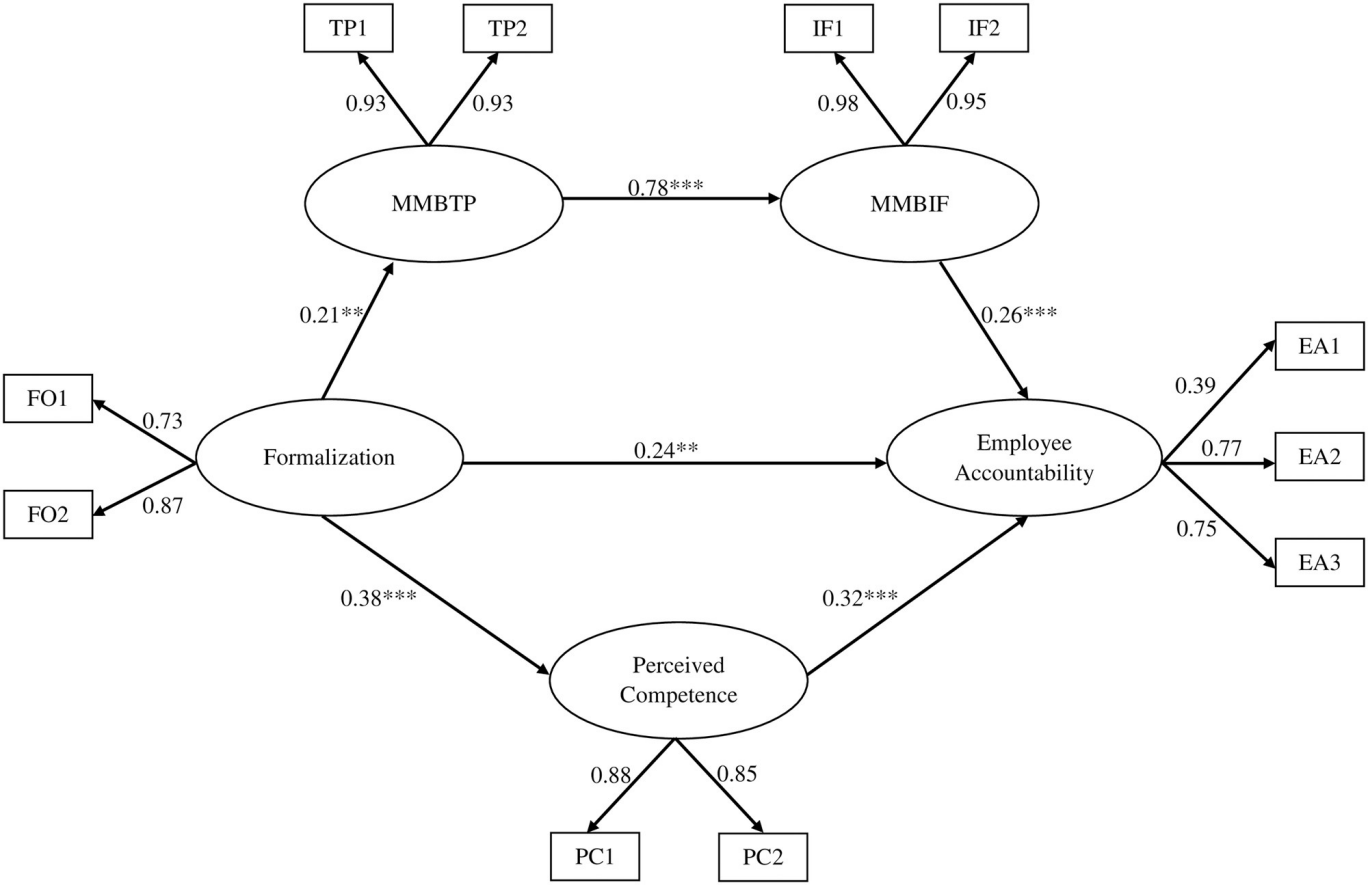
The mediating effect of managerial monitoring behavior and perceived competence on the relationship between formalization and employee accountability. MMBTP = Managerial monitoring behavior for task performance; MMBIF = Managerial monitoring behavior for interpersonal facilitation; TP1 = Managerial monitoring behavior for task performance 1; TP2 = Managerial monitoring behavior for task performance 2; IF1 = Managerial monitoring behavior for interpersonal facilitation 1; IF2 = Managerial monitoring behavior for interpersonal facilitation 2; FO1 = Formalization 1; FO2 = Formalization 2; PC1 = Perceived competence 1; PC2 = Perceived competence 2; EA1 = Employee accountability 1; EA2 = Employee accountability 2; EA3 = Employee accountability 3.

The results of hypothesis testing and the overall model are shown in Table 5 and Fig 2. In this research model, the job level has been controlled. Formalization has a significant positive relationship with employee accountability (*β* = 0.24, p < 0.01). This positive relationship supports hypothesis 1, which states that formalization is significantly and positively related to employee accountability. All indirect effects were tested using 5,000 bootstrap samples with a bootstrap confidence interval of 95%. The results of the analysis support the mediating effects of MMBTP and MMBIF (*β* = 0.01, p < 0.01, CI[0.01, 0.03]), indicating that hypothesis 2 is supported. The results of the analysis also support hypothesis 3, which assumes a mediating effect of perceived competence on the relationship between formalization and employee accountability (*β* = 0.04, p < 0.001, CI[0.02, 0.07]). The results of the analysis support all the hypotheses proposed in this study. In comparison, the direct effect of formalization and employee accountability is greater than the indirect effect mediated by both managerial monitoring behavior (task performance and interpersonal facilitation) and perceived competence. In the indirect effect between formalization and employee accountability, perceived competence shows a greater mediating effect than the mediating effect of managerial monitoring behavior.

**Table 5:** Squared multiple correlation and standardized total effect.

Variable	R^2^	Formalisasi	MMBTP	MMBIF	PC	Job Level
MMBTP	0.07	0.21**	-	-	-	0.13*
MMBIF	0.63	-	0.78***	-	-	0.06
PC	0.20	0.38***	-	-	-	0.19***
EA	0.33	0.24**	-	0.26***	0.32***	-0.02

* p< 0.05;

** p< 0.01;

*** p< 0.001;

MMBIF = Managerial monitoring behavior for interpersonal facilitation; MMBTP = Managerial monitoring behavior for task performance; PC = Perceived competence; EA = Employee accountability

## Discussion

The current study contributes to explaining the mechanism by which formalization affect accountability in a few different ways.

First, this study shows that in the mechanism of formalization and employee accountability, formalization has a greater direct effect than mediation. This result is in line with SCT, that indicates the importance of environment on behavior, suggesting the direct relationship between formalization and employee accountability. This study shows that high formalization in government agencies can establish employee accountability without the need for mediation. Therefore, practitioners may want to clarify rules, procedures, and regulations so that employees have a clearer understanding of the behaviors expected from them, and the expected evaluation. Having said the importance of practitioners to increase formalization, we need to remember that formalization may be an obstacle for employee creativity [36]. Future research need to reveal the optimal level of formalization that is important for employee accountability without limiting employee creativity.

Second, although the results of this study support a stronger direct relationship between formalization and employee accountability, the results also show significant mediating roles. The current study advances our understanding by demonstrating that formalization may actually increase employee accountability through managerial monitoring behavior (MMBTP and MMBIF) and perceived competence. This finding is important, as formalization is a central and classic challenge in government agencies [5] and leaders can assist in determining how to apply formalization to employees [24]. Knowing the mechanism by which how formalization influences employee accountability, organizations may focus not only on improving their formalization (i.e., procedures and regulations) but also on paying attention to the roles of leadership (i.e., managerial monitoring behavior) and individual aspects of employees (i.e., perceived competence).

Compared to managerial monitoring behavior, perceived competence plays a greater mediating role in the mechanism of formalization and employee accountability. This result is inline with SCT that argues how environment affect individuals and then in turn affect behavior. The results of the analysis of the mediating role of perceived competence are in accordance with the explanation of SCT, where the environment affects individuals and then, in turn, affects behavior. This is in line with Bartimore-Aufflick et al.’s [58] finding that regulation can increase perceived competence. Likewise, Chamberlin et al. [46] show that perceived competence boosts positive employees work behavior. This result may occur because of the special characteristics of the sample of this current study. The average age of the current study is 37.68, which is categorized as generation Y. This generation’s characteristics are tech entusiast, self-centered, ambitious, and want meaningful work [59]. The Y generation is distinguished by its high self-esteem, sense of entitlement, and self-centeredness [7]. This could explain why perceived competence has a greater influence as a mediator than managerial monitoring behavior. HR practitioners may want to focus on the micro-foundations of HRM, that is the individual employees to increase employee accountability. Specifically, HR practitioners may want to focus on the selection process, that is in choosing employees with a strong perceived competence. As well, employees need to be given the opportunity to show and improve their competence, as our research shows that perceived competence significantly mediated the relationship between formalization and employee accountability.

Third, this study shows that MMBTP and MMBIF serially mediate the relationship between formalization and employee accountability. In this study, the measured MMBIF is the employee’s perception of interpersonal supervision in the context of communication related to task implementation, so the high correlation indicates that MMBTP is a factor that initiates MMBIF. In a serial relationship, formalization as an environmental factor in SCT increases MMBTP in superiors, which in turn intensifies MMBIF. Supervisors’ MMBIF will then escalating employee accountability (work behavior). Supporting this research, a previous study conducted by Josephine and Riantoputra [25] in Indonesia showed a high correlation between MMBTP and MMBIF, while Mero et al.’s [17] study conducted in America showed an insignificant correlation. Cultural factors might explain the difference in the correlation between MMBTP and MMBIF in Indonesia and America, as cultural factors can influence how individuals perceive and process feedback, such as in the process of mentoring [60]. Further research may want to investigate cultural factor, such as high power distance, that potentially affect the way employees perceive monitoring behavior from their leaders. To enhance employee accountability through managerial monitoring behavior, leaders may need to help employees to understand the why behind their tasks and targets. Understanding the significance of these tasks and targets, may help employees to agree on them, and thus more willing to be accountable on those behaviors. One aspect of HRM that speaks heavily on accountability is performance appraisal, as it makes clear the expected behaviors and attitudes of employees and the rewards and sanctions related to those behaviors and attitudes. This is especially important as Carucci [2] states that 70% of employees feel their managers are not objective in how they evaluate their employees. HRM practitioners may want to provide training to empower leaders to do a better performance appraisal review.

The result reached in this study may make sense in governance agency setting in Indonesia. According to Cameron and Quinn’s [61] cultural value model, this organization type is part of the hierarchical culture. This type of culture shows a high formalization, and high control over work implementation. The findings of this study are consistent with the CVF because they focus on formalization and increasing employee accountability through serial managerial monitoring behavior (control), first through MMBTP (job supervision) and then through MMBIF (cooperation and relationship supervision). The study’s findings suggest that government organizations in Indonesia might also pay attention to leader and individual factors, as well as formalization in establishing employee accountability. Future research may wish to examine employee accountability in different organizational settings (e.g. start-up companies) to examine the relationship between formalization and employee accountability and other work behaviors.

Fourth, the current study advances our understanding of employee accountability by demonstrating that job level positively correlates with managerial monitoring behavior and perceived competence. Both Kong et al. [62] and Yang et al. [63] showed that job level influenced how individuals perceive their competence in China and Taiwan, respectively. According to Hofstede et al. [64], Indonesia, China, and Taiwan are regarded as countries with high power distance. This means that people in these countries tend to accept power inequality between leaders and their subordinates. Within this cultural context, leaders are highly respected and trusted by their subordinates. Thus, as employees advance to higher job levels, they will experience an increase in the level of trust that their subordinates place in them. Future research should recognize the impact of job level, especially in organizations with high power distance culture.

This study has several limitations. First, we used a self-report method to collect data from employees (i.e., single source), which could potentially produce common method bias. While this does not appear to be the case according to the results of Harman’s single-factor test, we contend that future research should consider employing multisource data or time-lagged data collection to meaningfully reduce common method bias [65]. Secondly, in this study, we specifically focused on organizations with high formalization; thus, our findings might not generalize to other organizations with different levels of formalization.

## Conclusion

This study advances international HRM literature by demonstrating a positive relationship between formalization and employee accountability mediated by managerial monitoring behavior and perceived competence. This study provides empirical support for the model of dynamic and reciprocal interaction between person, environments, and behavior (i.e., SCT). In this model, perceived competence (person component), formalization and managerial monitoring behavior (environment component), and employee accountability (behavior component) act as determinants that can influence each other, though not simultaneously. Moreover, this study highlights the importance of the interaction between leaders, employees, and organizational factors in exploring employee accountability within a socio-cognitive context.
