# Supplementary material for: Employee accountability in Indonesia: The role of formalization, managerial monitoring behavior and perceived competence
Source: PLoS One. 2022 Dec 16;17(12):e0278330. doi: 10.1371/journal.pone.0278330 (PMC9757594; doi:10.1371/journal.pone.0278330)
Supplement: Attachment — Submitted filename: Reviewer Recommendation and Comments for PONE.docx [file pone.0278330.s001.docx]

**Reviewer Recommendation and Comments for PONE-D-22-25665**

It is recommended to major revision. Based on the reality of a multi-population developing country like Indonesia, this article discusses the relationship between Formalization, Manager Monitoring Behavior, Perceived Competence, and Employee Accountability. It has certain practical significance. However, the structure layout, research design, demonstration analysis and format specifications of the manuscript need to be modified and improved.

1. Authors are asked to report the study's compliance with internationally accepted ethical norms and informed consent.

2. The structure layout of the manuscript needs to be further optimized. It is recommended to merge the introduction and Literature review. In the manuscript structures, Literature Review and Formalization, Manager Monitoring Behavior, Perceived Competence, and Employee Accountability are all separate paragraphs. The logical relationships look parallel, but this is actually not this relationship. What is the relationship between Literature review and Formalization, Manager Monitoring Behavior, Perceived Competence and Employee Accountability in the existing manuscript structure?

It is recommended that the relevant concepts should be concentrated a chapter, and discuss separately according to the category. Based on this above, put forward corresponding theoretical assumptions. Independent literature review and independent concept introduction chapter are not common.

3.The theoretical hypothesis model lacks the hypothetical relationship diagram of each variable, and it is recommended to supplement it. After theoretical assumptions, the lack of such a hypothetical diagram seems uncomfortable.

4. As shown in the variable effect diagram, combined with the literature review of Managerial monitoring behavior, the author omits the analysis of whether Formalization can have a direct impact on MMBIF. Judging from the existing literature, Formalization can not only promote the improvement of performance, but also produce changes in interpersonal emotions. The influence relationship between the two is parallel. Only the direct impact of Formalization on MMBTP is considered, while the direct impact on MMBIF is ignored. There are certain defects, and it is recommended to supplement.

5. The author introduces the composition of the scale, which is composed of five different mature scale parts. Although the reliability and validity of each component are introduced, the reliability and validity of the new integrated scale have not been elaborated and need to be supplemented.

6. In the results, Table 2 the correlation table needs to be clearly identified, and the variables that cannot be clearly indicated by the 1, 2, 3, 4, 5…10 symbols alone. Table 3 does not conform to the marking specifications for annotations on commonly used three-line tables.

7 What is the relationship between the practical implications and discussion? Some contents overlap with discussions, such as MMBTP, MMBIF, and the relationship of mediating role between the formalization and employee responsibility.

It is recommended to put the Practical implications as a part of the discussion in the discussion.

8. In the discussion, the second and third points discussed the mediating role of MMBTP and MMBIF ，the important role of the two variables. There are some repetitions in logic and content. Is there any purpose for the author to arrange this? It is recommended that the discussion of mediation and effects of these two variables be grouped together.

9. In the discussion, the more direct effect of Formalization and the related discussions about the mediating role of MMBTP and MMBIF were mainly analyzed in the theoretical literature.

Without considering the particularities of Indonesian society and the actual working environment, it is impossible to explain that these conclusions are unique to Indonesia? Or can it also happen in other countries? What are the reasons for this in the working environment in Indonesia.

10.The implications of the findings for Indonesian society and the real-world work environment were not presented in the Discussion and Conclusions. This needs to be added.

11. The discussion of the mediating role of perception is scattered among the various points of discussion. Please form a paragraph to focus on the mediating role of perception, explain the process of effect generation, the difference between the mediating role of MMBTP and MMBIF.
