# Supplementary material for: Employee accountability in Indonesia: The role of formalization, managerial monitoring behavior and perceived competence
Source: PLoS One. 2022 Dec 16;17(12):e0278330. doi: 10.1371/journal.pone.0278330 (PMC9757594; doi:10.1371/journal.pone.0278330)
Supplement: Attachment — Submitted filename: Response to Reviewer PLOS one.docx [file pone.0278330.s002.docx]

4^th^ Nov 2022

Emily Chenette

Editor-in-chief

*PLOS One*

Dear Chenette,

We greatly appreciate the reviewers for their thoughtful and insightful comments. We have thoroughly revised our manuscript in response to their suggestions and believe that the manuscript has improved significantly because of that. We made some significant changes in the introduction, methods, discussion and practical implications. Please see below for our detailed response to address each comment.

1. Authors are asked to report the study's compliance with internationally accepted ethical norms and informed consent.

- Thank you for your input. We have added the ethical approval to inform the ethical compliance and informed consent of this study (see page 14).

2. The structure layout of the manuscript needs to be further optimized. It is recommended to merge the introduction and Literature review. In the manuscript structures, Literature Review and Formalization, Manager Monitoring Behavior, Perceived Competence, and Employee Accountability are all separate paragraphs. The logical relationships look parallel, but this is actually not this relationship. What is the relationship between Literature review and Formalization, Manager Monitoring Behavior, Perceived Competence and Employee Accountability in the existing manuscript structure?

It is recommended that the relevant concepts should be concentrated a chapter, and discuss separately according to the category. Based on this above, put forward corresponding theoretical assumptions. Independent literature review and independent concept introduction chapter are not common.

- We really appreciate your significant recommendations. In response to this, we have revised the structure by combining introduction and literature review, and by discussing each relationship as one subsection. That is, we discussed formalization and employee accountability, managerial monitoring behavior as mediator, and perceived competence as mediator as separate subsections (see page 2 – 12).

3. The theoretical hypothesis model lacks the hypothetical relationship diagram of each variable, and it is recommended to supplement it. After theoretical assumptions, the lack of such a hypothetical diagram seems uncomfortable

- Thank you for your practical yet useful input. We have added the hypothetical relationship diagram accordingly (see page 8).

4. As shown in the variable effect diagram, combined with the literature review of Managerial monitoring behavior, the author omits the analysis of whether Formalization can have a direct impact on MMBIF. Judging from the existing literature, Formalization can not only promote the improvement of performance, but also produce changes in interpersonal emotions. The influence relationship between the two is parallel. Only the direct impact of Formalization on MMBTP is considered, while the direct impact on MMBIF is ignored. There are certain defects, and it is recommended to supplement.

- We appreciate your insightful comment that help us to revise our manuscript significantly. In our revised manuscript, we have made it clearer that one of the research gap we aim to fill in is the mechanism by which formalization influences employee accountability. That is, differ from previous research, we argue that MMBTP and MMBIF mediate the relationship between formalization and employee accountability, serially. We have added more arguments and references to support our position. (see research gap number 3 on page 5 and hypothesis number 2 on page 10 – 11). We hope that you can see that our data set supports our position and thus our research has contributed to the knowledge of employee accountability.

5. The author introduces the composition of the scale, which is composed of five different mature scale parts. Although the reliability and validity of each component are introduced, the reliability and validity of the new integrated scale have not been elaborated and need to be supplemented.

- Thank you for your useful direction. We took your input seriously and have taken several steps to show the reliability of each scale and further scale as a whole. We have added CFA analysis, composite reliability, and Average Variance Extracted (AVE) (see page 16 – 17).

6. In the results, Table 2 the correlation table needs to be clearly identified, and the variables that cannot be clearly indicated by the 1, 2, 3, 4, 5…10 symbols alone. Table 3 does not conform to the marking specifications for annotations on commonly used three-line tables.

- Thank you for your suggestion. We have revised our table accordingly (see page 18 – 19 and 21).

7. What is the relationship between the practical implications and discussion? Some contents overlap with discussions, such as MMBTP, MMBIF, and the relationship of mediating role between the formalization and employee responsibility.

It is recommended to put the Practical implications as a part of the discussion in the discussion.

- We highly appreciate this recommendation. In response, we have moved practical implication right after the discussion related to theoretical advancement. We have also deleted some of the practical implication that are not directly related to our research results. By so doing, the quality of our discussion section has improved significantly and we thank you for that (see page 21 – 25).

8. In the discussion, the second and third points discussed the mediating role of MMBTP and MMBIF, the important role of the two variables. There are some repetitions in logic and content. Is there any purpose for the author to arrange this? It is recommended that the discussion of mediation and effects of these two variables be grouped together.

- Thank you for your input. As it is explained in point seven, we have rearranged the discussion section to reduce any repetition and to increase logic and substantive arguments (see page 23 – 24).

9. In the discussion, the more direct effect of Formalization and the related discussions about the mediating role of MMBTP and MMBIF were mainly analyzed in the theoretical literature.

Without considering the particularities of Indonesian society and the actual working environment, it is impossible to explain that these conclusions are unique to Indonesia? Or can it also happen in other countries? What are the reasons for this in the working environment in Indonesia?

- We really appreciate your insightful comment that make us think the particularities in Indonesian society and the research setting. Therefore, we have added a new paragraph to elaborate this aspect and thus help readers to see the uniqueness of the research result. We also have added some sentences to call for more research on this aspect but in the different research setting (see page 24 -25).

10. The implications of the findings for Indonesian society and the real-world work environment were not presented in the Discussion and Conclusions. This needs to be added.

- Thank you for your suggestion. As we have explained in point nine, we have added more discussion on implication on this finding for the Indonesian society (see page 24 – 25).

11. The discussion of the mediating role of perception is scattered among the various points of discussion. Please form a paragraph to focus on the mediating role of perception, explain the process of effect generation, the difference between the mediating role of MMBTP and MMBIF.

- We appreciate this comment. As you can see on page 23, we have rearranged all discussion related to the mediating role of perceived competence in one paragraph.

Hope you agree with us, that we have taken your input seriously and have put considerable effort in revising our manuscript.
