# Supplementary material for: Employee accountability in Indonesia: The role of formalization, managerial monitoring behavior and perceived competence
Source: PLoS One. 2022 Dec 16;17(12):e0278330. doi: 10.1371/journal.pone.0278330 (PMC9757594; doi:10.1371/journal.pone.0278330)
Supplement: Peer review history

# Peer review: Decision Letter 0

**DOI:** 10.1371/journal.pone.0278330.r001

**Authors:** Rogis Baker

14 Oct 2022

PONE-D-22-25665Employee accountability in Indonesia: The role of formalization, managerial monitoring behavior and perceived competencePLOS ONE

Dear Dr. Corina D Riantoputra,

Thank you for submitting your manuscript to PLOS ONE. After careful consideration, we feel that it has merit but does not fully meet PLOS ONE’s publication criteria as it currently stands. Therefore, we invite you to submit a revised version of the manuscript that addresses the points raised during the review process.

Please submit your revised manuscript by October 21, 2022. If you will need more time than this to complete your revisions, please reply to this message or contact the journal office at plosone@plos.org. When you're ready to submit your revision, log on to https://www.editorialmanager.com/pone/ and select the 'Submissions Needing Revision' folder to locate your manuscript file.

Please include the following items when submitting your revised manuscript:

- A rebuttal letter that responds to each point raised by the academic editor and reviewer(s). You should upload this letter as a separate file labeled 'Response to Reviewers'.
- A marked-up copy of your manuscript that highlights changes made to the original version. You should upload this as a separate file labeled 'Revised Manuscript with Track Changes'.
- An unmarked version of your revised paper without tracked changes. You should upload this as a separate file labeled 'Manuscript'.

If you would like to make changes to your financial disclosure, please include your updated statement in your cover letter. Guidelines for resubmitting your figure files are available below the reviewer comments at the end of this letter.

If applicable, we recommend that you deposit your laboratory protocols in protocols.io to enhance the reproducibility of your results. Protocols.io assigns your protocol its own identifier (DOI) so that it can be cited independently in the future. For instructions see: https://journals.plos.org/plosone/s/submission-guidelines#loc-laboratory-protocols. Additionally, PLOS ONE offers an option for publishing peer-reviewed Lab Protocol articles, which describe protocols hosted on protocols.io. Read more information on sharing protocols at https://plos.org/protocols?utm_medium=editorial-email&utm_source=authorletters&utm_campaign=protocols.

We look forward to receiving your revised manuscript.

Kind regards,

Rogis Baker, Ph.D

Academic Editor

PLOS ONE

Journal Requirements:

When submitting your revision, we need you to address these additional requirements.

1. Please ensure that your manuscript meets PLOS ONE's style requirements, including those for file naming. The PLOS ONE style templates can be found at 

https://journals.plos.org/plosone/s/file?id=wjVg/PLOSOne_formatting_sample_main_body.pdf and 

https://journals.plos.org/plosone/s/file?id=ba62/PLOSOne_formatting_sample_title_authors_affiliations.pdf

2. Please provide additional details regarding ethical approval and participant consent in the body of your manuscript. In the Methods section, please ensure that you have specified (1) the name of the IRB/ethics committee that approved your study, (2) whether consent was informed and (3) what type of consent you obtained (for instance, written or verbal). If your study included minors, state whether you obtained consent from parents or guardians. If the need for consent was waived by the ethics committee, please include this information.

3. We note that you have stated that you will provide repository information for your data at acceptance. Should your manuscript be accepted for publication, we will hold it until you provide the relevant accession numbers or DOIs necessary to access your data. If you wish to make changes to your Data Availability statement, please describe these changes in your cover letter and we will update your Data Availability statement to reflect the information you provide.

4. Please amend either the abstract on the online submission form (via Edit Submission) or the abstract in the manuscript so that they are identical.

5. Please include your full ethics statement in the ‘Methods’ section of your manuscript file. In your statement, please include the full name of the IRB or ethics committee who approved or waived your study, as well as whether or not you obtained informed written or verbal consent. If consent was waived for your study, please include this information in your statement as well. 

[Note: HTML markup is below. Please do not edit.]

Reviewers' comments:

Reviewer's Responses to Questions

**Comments to the Author**

1. Is the manuscript technically sound, and do the data support the conclusions?

The manuscript must describe a technically sound piece of scientific research with data that supports the conclusions. Experiments must have been conducted rigorously, with appropriate controls, replication, and sample sizes. The conclusions must be drawn appropriately based on the data presented. 

Reviewer #1: Partly

Reviewer #2: Yes

Reviewer #3: Yes

**********

2. Has the statistical analysis been performed appropriately and rigorously? 

Reviewer #1: Yes

Reviewer #2: Yes

Reviewer #3: Yes

**********

3. Have the authors made all data underlying the findings in their manuscript fully available?

The PLOS Data policy requires authors to make all data underlying the findings described in their manuscript fully available without restriction, with rare exception (please refer to the Data Availability Statement in the manuscript PDF file). The data should be provided as part of the manuscript or its supporting information, or deposited to a public repository. For example, in addition to summary statistics, the data points behind means, medians and variance measures should be available. If there are restrictions on publicly sharing data—e.g. participant privacy or use of data from a third party—those must be specified.

Reviewer #1: Yes

Reviewer #2: Yes

Reviewer #3: Yes

**********

4. Is the manuscript presented in an intelligible fashion and written in standard English?

PLOS ONE does not copyedit accepted manuscripts, so the language in submitted articles must be clear, correct, and unambiguous. Any typographical or grammatical errors should be corrected at revision, so please note any specific errors here.

Reviewer #1: Yes

Reviewer #2: Yes

Reviewer #3: Yes

**********

5. Review Comments to the Author

Please use the space provided to explain your answers to the questions above. You may also include additional comments for the author, including concerns about dual publication, research ethics, or publication ethics. (Please upload your review as an attachment if it exceeds 20,000 characters)

Reviewer #1: It is recommended to major revision. Based on the reality of a multi-population developing country like Indonesia, this article discusses the relationship between Formalization, Manager Monitoring Behavior, Perceived Competence, and Employee Accountability. It has certain practical significance. However, the structure layout, research design, demonstration analysis and format specifications of the manuscript need to be modified and improved.

1. Authors are asked to report the study's compliance with internationally accepted ethical norms and informed consent.

2. The structure layout of the manuscript needs to be further optimized. It is recommended to merge the introduction and Literature review. In the manuscript structures, Literature Review and Formalization, Manager Monitoring Behavior, Perceived Competence, and Employee Accountability are all separate paragraphs. The logical relationships look parallel, but this is actually not this relationship. What is the relationship between Literature review and Formalization, Manager Monitoring Behavior, Perceived Competence and Employee Accountability in the existing manuscript structure?

It is recommended that the relevant concepts should be concentrated a chapter, and discuss separately according to the category. Based on this above, put forward corresponding theoretical assumptions. Independent literature review and independent concept introduction chapter are not common.

3.The theoretical hypothesis model lacks the hypothetical relationship diagram of each variable, and it is recommended to supplement it. After theoretical assumptions, the lack of such a hypothetical diagram seems uncomfortable.

4. As shown in the variable effect diagram, combined with the literature review of Managerial monitoring behavior, the author omits the analysis of whether Formalization can have a direct impact on MMBIF. Judging from the existing literature, Formalization can not only promote the improvement of performance, but also produce changes in interpersonal emotions. The influence relationship between the two is parallel. Only the direct impact of Formalization on MMBTP is considered, while the direct impact on MMBIF is ignored. There are certain defects, and it is recommended to supplement.

5. The author introduces the composition of the scale, which is composed of five different mature scale parts. Although the reliability and validity of each component are introduced, the reliability and validity of the new integrated scale have not been elaborated and need to be supplemented.

6. In the results, Table 2 the correlation table needs to be clearly identified, and the variables that cannot be clearly indicated by the 1, 2, 3, 4, 5…10 symbols alone. Table 3 does not conform to the marking specifications for annotations on commonly used three-line tables.

7. What is the relationship between the practical implications and discussion? Some contents overlap with discussions, such as MMBTP, MMBIF, and the relationship of mediating role between the formalization and employee responsibility.

It is recommended to put the Practical implications as a part of the discussion in the discussion.

8. In the discussion, the second and third points discussed the mediating role of MMBTP and MMBIF ，the important role of the two variables. There are some repetitions in logic and content. Is there any purpose for the author to arrange this? It is recommended that the discussion of mediation and effects of these two variables be grouped together.

9. In the discussion, the more direct effect of Formalization and the related discussions about the mediating role of MMBTP and MMBIF were mainly analyzed in the theoretical literature.

Without considering the particularities of Indonesian society and the actual working environment, it is impossible to explain that these conclusions are unique to Indonesia? Or can it also happen in other countries? What are the reasons for this in the working environment in Indonesia.

10. The implications of the findings for Indonesian society and the real-world work environment were not presented in the Discussion and Conclusions. This needs to be added.

11. The discussion of the mediating role of perception is scattered among the various points of discussion. Please form a paragraph to focus on the mediating role of perception, explain the process of effect generation, the difference between the mediating role of MMBTP and MMBIF.

Reviewer #2: Notes From Reviewers

1. Summary and overall impression

Overall this article manuscript is quite good because it is presented in a communicative language. It's just that the author uses a lot of references to old theories, so it doesn't seem up to date anymore. In addition, the author failed to reveal the novelty of his research. The author presents many relevant research results, but fails to point out the weaknesses of the previous research. It was reported from the results of previous studies that several social problems emerged in the budgeting process for the management of public institutions, but did not explicitly state what the social problems were. It is stated that management behavior and the role of acting as an agent and structure that oversees the implementation process to actualize the budgeting process effectively and efficiently, but the results of previous studies that support this sentence are not presented. In addition, it is not clear why the author is interested in analyzing budget formulation in a social context.

The reviewers argue that the author should use a reference to a theory that is relatively new. Then the writer should try to reveal the novelty of his research by presenting various relevant previous research results, especially those related to social problems that arise in the budgeting process of public institutions. Likewise, there needs to be research results that support the statement that management behavior and the role of acting as an agent and structure that oversees the implementation process to actualize the budgeting process effectively and efficiently because this statement is not a tautology. The author also needs to point out the urgency of analyzing budget formulation in a social context.

2. Discussion of specific areas for improvement

This research uses Structural Theory from Anthony Giddens. The author has analyzed the results of research conducted using structuration theory by dividing it into three parts, namely the strength of the agent (see page 15), the strength of the structure (see page 21), and the interaction between the agent and the structure (see page 22). The author has elaborated well, but the analysis carried out does not explicitly link the three elements in the structuration theory as stated by the author on page 12, namely: 1). rationalization which is represented in seeking security and comfort, 2) motivation as a desire that drives action, and 3) consciousness, divided into discursive consciousness which represents action and practical consciousness which represents in its truest essence. It is better if the analysis of the discussion carried out represents these three things.

Reviewer #3: This is a very well designed based study and very well written. The hypothesis are very clear and addressed appropriately.

There are few editorial errors such a "undertand" instead of "understand" on page 2.

I suggest to improve the quality of the figure.

**********

6. PLOS authors have the option to publish the peer review history of their article (what does this mean?). If published, this will include your full peer review and any attached files.

If you choose “no”, your identity will remain anonymous but your review may still be made public.

**Do you want your identity to be public for this peer review?** For information about this choice, including consent withdrawal, please see our Privacy Policy.

Reviewer #1: No

Reviewer #2: No

Reviewer #3: No

**********

[NOTE: If reviewer comments were submitted as an attachment file, they will be attached to this email and accessible via the submission site. Please log into your account, locate the manuscript record, and check for the action link "View Attachments". If this link does not appear, there are no attachment files.]

While revising your submission, please upload your figure files to the Preflight Analysis and Conversion Engine (PACE) digital diagnostic tool, https://pacev2.apexcovantage.com/. PACE helps ensure that figures meet PLOS requirements. To use PACE, you must first register as a user. Registration is free. Then, login and navigate to the UPLOAD tab, where you will find detailed instructions on how to use the tool. If you encounter any issues or have any questions when using PACE, please email PLOS at figures@plos.org. Please note that Supporting Information files do not need this step.

Attachment Submitted filename: Reviewer Recommendation and Comments for PONE.docx Click here for additional data file.

# Author comment: Author response to Decision Letter 0

**DOI:** 10.1371/journal.pone.0278330.r002

8 Nov 2022

We greatly appreciate the reviewers for their thoughtful and insightful comments. We have thoroughly revised our manuscript in response to their suggestions and believe that the manuscript has improved significantly because of that. We made some significant changes in the introduction, methods, discussion and practical implications. Please see below for our detailed response to address each comment. 

1. Authors are asked to report the study's compliance with internationally accepted ethical norms and informed consent. 

• Thank you for your input. We have added the ethical approval to inform the ethical compliance and informed consent of this study (see page 14).

2. The structure layout of the manuscript needs to be further optimized. It is recommended to merge the introduction and Literature review. In the manuscript structures, Literature Review and Formalization, Manager Monitoring Behavior, Perceived Competence, and Employee Accountability are all separate paragraphs. The logical relationships look parallel, but this is actually not this relationship. What is the relationship between Literature review and Formalization, Manager Monitoring Behavior, Perceived Competence and Employee Accountability in the existing manuscript structure?

It is recommended that the relevant concepts should be concentrated a chapter, and discuss separately according to the category. Based on this above, put forward corresponding theoretical assumptions. Independent literature review and independent concept introduction chapter are not common.

• We really appreciate your significant recommendations. In response to this, we have revised the structure by combining introduction and literature review, and by discussing each relationship as one subsection. That is, we discussed formalization and employee accountability, managerial monitoring behavior as mediator, and perceived competence as mediator as separate subsections (see page 2 – 12).

3. The theoretical hypothesis model lacks the hypothetical relationship diagram of each variable, and it is recommended to supplement it. After theoretical assumptions, the lack of such a hypothetical diagram seems uncomfortable

• Thank you for your practical yet useful input. We have added the hypothetical relationship diagram accordingly (see page 8). 

4. As shown in the variable effect diagram, combined with the literature review of Managerial monitoring behavior, the author omits the analysis of whether Formalization can have a direct impact on MMBIF. Judging from the existing literature, Formalization can not only promote the improvement of performance, but also produce changes in interpersonal emotions. The influence relationship between the two is parallel. Only the direct impact of Formalization on MMBTP is considered, while the direct impact on MMBIF is ignored. There are certain defects, and it is recommended to supplement.

• We appreciate your insightful comment that help us to revise our manuscript significantly. In our revised manuscript, we have made it clearer that one of the research gap we aim to fill in is the mechanism by which formalization influences employee accountability. That is, differ from previous research, we argue that MMBTP and MMBIF mediate the relationship between formalization and employee accountability, serially. We have added more arguments and references to support our position. (see research gap number 3 on page 5 and hypothesis number 2 on page 10 – 11). We hope that you can see that our data set supports our position and thus our research has contributed to the knowledge of employee accountability. 

5. The author introduces the composition of the scale, which is composed of five different mature scale parts. Although the reliability and validity of each component are introduced, the reliability and validity of the new integrated scale have not been elaborated and need to be supplemented.

• Thank you for your useful direction. We took your input seriously and have taken several steps to show the reliability of each scale and further scale as a whole. We have added CFA analysis, composite reliability, and Average Variance Extracted (AVE) (see page 16 – 17). 

6. In the results, Table 2 the correlation table needs to be clearly identified, and the variables that cannot be clearly indicated by the 1, 2, 3, 4, 5…10 symbols alone. Table 3 does not conform to the marking specifications for annotations on commonly used three-line tables.

• Thank you for your suggestion. We have revised our table accordingly (see page 18 – 19 and 21). 

7. What is the relationship between the practical implications and discussion? Some contents overlap with discussions, such as MMBTP, MMBIF, and the relationship of mediating role between the formalization and employee responsibility. 

It is recommended to put the Practical implications as a part of the discussion in the discussion.

• We highly appreciate this recommendation. In response, we have moved practical implication right after the discussion related to theoretical advancement. We have also deleted some of the practical implication that are not directly related to our research results. By so doing, the quality of our discussion section has improved significantly and we thank you for that (see page 21 – 25). 

8. In the discussion, the second and third points discussed the mediating role of MMBTP and MMBIF, the important role of the two variables. There are some repetitions in logic and content. Is there any purpose for the author to arrange this? It is recommended that the discussion of mediation and effects of these two variables be grouped together.

• Thank you for your input. As it is explained in point seven, we have rearranged the discussion section to reduce any repetition and to increase logic and substantive arguments (see page 23 – 24).

9. In the discussion, the more direct effect of Formalization and the related discussions about the mediating role of MMBTP and MMBIF were mainly analyzed in the theoretical literature.

Without considering the particularities of Indonesian society and the actual working environment, it is impossible to explain that these conclusions are unique to Indonesia? Or can it also happen in other countries? What are the reasons for this in the working environment in Indonesia?

• We really appreciate your insightful comment that make us think the particularities in Indonesian society and the research setting. Therefore, we have added a new paragraph to elaborate this aspect and thus help readers to see the uniqueness of the research result. We also have added some sentences to call for more research on this aspect but in the different research setting (see page 24 -25).

10. The implications of the findings for Indonesian society and the real-world work environment were not presented in the Discussion and Conclusions. This needs to be added.

• Thank you for your suggestion. As we have explained in point nine, we have added more discussion on implication on this finding for the Indonesian society (see page 24 – 25). 

11. The discussion of the mediating role of perception is scattered among the various points of discussion. Please form a paragraph to focus on the mediating role of perception, explain the process of effect generation, the difference between the mediating role of MMBTP and MMBIF.

• We appreciate this comment. As you can see on page 23, we have rearranged all discussion related to the mediating role of perceived competence in one paragraph. 

Hope you agree with us, that we have taken your input seriously and have put considerable effort in revising our manuscript.

Attachment Submitted filename: Response to Reviewer PLOS one.docx Click here for additional data file.

# Editor report: Decision Letter 1

**DOI:** 10.1371/journal.pone.0278330.r003

**Authors:** Rogis Baker

15 Nov 2022

Employee accountability in Indonesia: The role of formalization, managerial monitoring behavior and perceived competence

PONE-D-22-25665R1

Dear Dr. Corina D Riantoputra,

We’re pleased to inform you that your manuscript has been judged scientifically suitable for publication and will be formally accepted for publication once it meets all outstanding technical requirements.

Within one week, you’ll receive an e-mail detailing the required amendments. When these have been addressed, you’ll receive a formal acceptance letter and your manuscript will be scheduled for publication.

An invoice for payment will follow shortly after the formal acceptance. To ensure an efficient process, please log into Editorial Manager at http://www.editorialmanager.com/pone/, click the 'Update My Information' link at the top of the page, and double check that your user information is up-to-date. If you have any billing related questions, please contact our Author Billing department directly at authorbilling@plos.org.

If your institution or institutions have a press office, please notify them about your upcoming paper to help maximize its impact. If they’ll be preparing press materials, please inform our press team as soon as possible -- no later than 48 hours after receiving the formal acceptance. Your manuscript will remain under strict press embargo until 2 pm Eastern Time on the date of publication. For more information, please contact onepress@plos.org.

Kind regards,

Rogis Baker, Ph.D

Academic Editor

PLOS ONE

Additional Editor Comments (optional):

Reviewers' comments:

# Editor report: Acceptance letter

**DOI:** 10.1371/journal.pone.0278330.r004

**Authors:** Rogis Baker

8 Dec 2022

PONE-D-22-25665R1 

Employee accountability in Indonesia: The role of formalization, managerial monitoring behavior and perceived competence 

Dear Dr. Riantoputra:

I'm pleased to inform you that your manuscript has been deemed suitable for publication in PLOS ONE. Congratulations! Your manuscript is now with our production department. 

If your institution or institutions have a press office, please let them know about your upcoming paper now to help maximize its impact. If they'll be preparing press materials, please inform our press team within the next 48 hours. Your manuscript will remain under strict press embargo until 2 pm Eastern Time on the date of publication. For more information please contact onepress@plos.org.

If we can help with anything else, please email us at plosone@plos.org. 

Thank you for submitting your work to PLOS ONE and supporting open access. 

Kind regards, 

PLOS ONE Editorial Office Staff

on behalf of

Dr. Rogis Baker 

Academic Editor

PLOS ONE
